# Global patterns and drivers of species and genera richness of Fabaceae

**DOI:** 10.3389/fpls.2025.1581814

**Published:** 2025-07-21

**Authors:** Sazada Siddiqui

**Affiliations:** Department of Biology, College of Science, King Khalid University, Abha, Saudi Arabia

**Keywords:** Fabaceae, species richness, global patterns, drivers, biogeography

## Abstract

The Fabaceae, a highly diverse and ecologically vital plant family, thrives across diverse biomes with remarkable nitrogen-fixation potential and functional adaptability. Despite its key role in global carbon and nitrogen cycles, the biogeographic patterns and environmental drivers of this important family remain understudied compared to other major angiosperm families. Here, we consolidate and curate a global dataset comprising 27,421 taxa of Fabaceae obtained from the World Checklist of Vascular Plants (WCVP) and the Global Inventory of Floras and Traits (GIFT) to investigate the geographical patterns of species and genera richness and their environmental determinants. Using generalized linear models with a negative binomial approach and hierarchical partitioning analysis, we assessed the influence of climatic, geographic, and topographic predictors derived from WorldClim and PaleoClim on the species and genera richness of Fabaceae. The results reveal heterogeneous patterns of species and genera richness of Fabaceae, with maximum richness centers in tropical regions, particularly in seasonally dry tropical biomes, followed by temperate and subtropical biomes. Across the globe, Southern America turns out to be the dominant source of this botanical family, followed by Africa and Asia-Temperate. The results also reveal unequal representation of species belonging to different biomes in different continents. I found different sets of climatic and geographic drivers that shape the taxonomic levels of Fabaceae across countries, with a maximum contribution of elevation range, temperature diurnal range, precipitation seasonality, annual mean temperature, temperature seasonality, and geographical area. The findings provide data-based evidence of climatic variability and topographic heterogeneity in influencing the patterns of species and genera richness by enhancing niche differentiation and microhabitat diversity. The results concur with the latitudinal diversity gradient and the tropical conservatism hypothesis, which posits that stable tropical environments promote high species diversification and persistence. The present study will serve as a model to be replicated in other families to bridge the existing knowledge gaps. Furthermore, the findings of this study will aid in understanding the ecological adaptations of Fabaceae, which have immediate implications for ecological restoration and sustainable management strategies.

## Introduction

The temporal and spatial structure of global plant species distribution and identifying the primary climatic and environmental drivers that drive these distributional trends comprise key quests in biogeography and macroecology, with substantial implications for biodiversity conservation and habitat restoration (Hagen et al., 2021; Xu et al., 2023). The gaps in understanding the taxonomic, biogeographic, and evolutionary aspects of global biodiversity must be addressed immediately and punctually to provide a reliable assessment of its current state and to improve the predictions of potential future changes (Hortal et al., 2015; Diniz Filho et al., 2023). Vascular plants, which include more than 340,000 species (Govaerts et al., 2021), play an important role in terrestrial ecosystems by sustaining ecological activities (Zhang et al., 2023) and providing crucial ecosystem services (Heino et al., 2021; Le Provost et al., 2023). A wide variety of morphological and ecological traits of the angiosperms justifies that this is the predominant plant group in the world (Benton et al., 2022; Helmstetter et al., 2023). However, species diversity within the angiosperm clade is unevenly distributed across geographic regions of the world (Smith et al., 2011; Qian et al., 2023). Determining the processes underlying this extraordinary diversity is still a significant biological challenge and a topic of great scientific interest (Cadotte et al., 2011; Keil and Chase, 2019). Understanding the spatial distribution and underlying potential drivers of this essential component of global plant biodiversity is essential for its effective management and conservation (Meyer et al., 2015; Qiao et al., 2023).

Species richness and diversity dynamics are assessed using alpha (α-diversity) and beta (β-diversity) indices, which quantify species accumulation and turnover across spatial scales (Crist et al., 2003; Baselga, 2010). The distributions and richness patterns of plant species across the globe are substantially influenced by habitat filtering and bioclimatic factors, especially temperature and precipitation (Kreft and Jetz, 2007; Testolin et al., 2021; Oyebanji et al., 2023; Cai et al., 2023, 2024). However, the proportional importance of these factors depends on regional context, taxonomic group, and spatial scale (Weigelt et al., 2015; Testolin et al., 2021). Analyzing the global distribution patterns and environmental drivers of dominant plant families is fundamental to unravelling the processes that have shaped the establishment and diversification of flowering plants (Roeble et al., 2024). Identifying the patterns and predictors of species richness for distinct clades or specific groups and elucidating variation in species richness across biogeographic realms and biomes along latitudinal gradients are critical for advancing botanical, ecological, evolutionary, and conservation science (Chartier et al., 2021; Sabatini et al., 2022). Different plant families exhibit distinct functional traits and evolutionary histories, influencing their global biogeographic patterns and environmental drivers (Huang et al., 2021; Tian et al., 2024). Variations in traits such as life history strategies, photosynthetic pathways, and reproductive mechanisms affect how plant families adapt to specific climates, soil types, and ecological interactions, leading to unique distribution patterns worldwide (Friedman, 2020; Hayes et al., 2021; Tian et al., 2024).

For several plant families, species richness patterns along latitudinal gradients have been explained by a number of hypotheses; nevertheless, for many taxa, these patterns are still not fully understood. For instance, Vitt et al. (2023) investigated the global patterns of taxonomic and phylogenetic endemism coupled with the evolutionary distinctiveness of the Orchidaceae. Similarly, Roeble et al. (2024) provided key insights into the island biogeography of the Asteraceae family. Likewise, Tian et al. (2024) combined phylogenomic, environmental ordination, and macroevolutionary approaches to investigate the evolutionary processes responsible for the high species diversity within and across temperate biomes in the cosmopolitan angiosperm family Rhamnaceae. Yang et al. (2022) examined how climatic factors, soil properties, and dispersal mechanisms influence the species richness of *Medicago* L.genus. Tietje et al. (2022) found that global species richness and diversification rates are unrelated, with the highest diversification occurring in dry, edaphically diverse regions affected by Neogene climate change, highlighting climate and environmental heterogeneity as key drivers of species richness. Moreover, Tietje et al. (2023) examined global patterns of plant species richness and phylogenetic diversity, and reported that while species richness peaks predominantly in the Neotropics, phylogenetic diversity is more evenly distributed across the globe than species richness, with tropical rainforests playing a crucial role in maintaining elevated levels of phylogenetic diversity. Although global studies have improved our understanding on the diversity and distribution of plants across globe, substantial knowledge gaps persist regarding the role of ecological interactions and niche conservatism in shaping diversity distribution across spatial and environmental gradients in various plant families (Vitt et al., 2023). Advancing macroecological assessments is critical for elucidating the drivers of species richness, predicting biodiversity responses to environmental changes, and informing conservation and land management strategies to sustain the ecological and economic functions of plant families (Nanglu et al., 2023; Wang et al., 2024; Roeble et al., 2024).

Fabaceae, also known as the Leguminosae family, is one of the largest and most ecologically important angiosperm family, comprising approximately 27,421 taxa distributed across diverse ecosystems worldwide (POWO, 2025). The family accounts for approximately 8% of global vascular plant species (Govaerts et al., 2021). This family is the third-largest among the angiosperms in terms of species numbers after Asteraceae and Orchidaceae (Lewis et al., 2005; POWO, 2025). The family displays an astounding diversity in morphology and habit with species ranging from arid shrubs, ephemeral herbs, and herbaceous climbers to massive rainforest trees and woody lianas (Lewis et al., 2005). Since its origin, approximately 67 million years ago, close to the Cretaceous/Paleogene boundary (Zhao et al., 2021), the family has undergone significant diversification, inhabiting environments that range from temperate woodlands and tropical rainforests to arid deserts and alpine regions (Lavin et al., 2005; Lewis et al., 2005). The family is essential to ecosystems worldwide, especially because of its capacity to fix nitrogen from the atmosphere through symbiotic partnershipssuch as *Rhizobia*bacteria, which improve soil fertility and sustain plant populations (Zhao et al., 2021). This ecological function makes legumes vital for agriculture, as many species, such as *Glycine max* (L.) Merr. (soybean), *Phaseolus vulgaris* L. (common bean), *Cajanus cajan* (L.) Huth(pigeon pea) and *Arachis hypogaea* L. (peanut) serve as major food and fodder crops worldwide (Lewis et al., 2005). Despite some attempts to comprehend the global patterns of species richness of various Fabaceae tribes, there are still a number of taxonomical, ecological and biogeographical shortfalls that hinder our progress toward accomplishing global conservation and sustainability objectives. Oyebanji et al. (2023) demonstrated that megathermal regions serve as hotspots of species richness for the Millettioid/Phaseoloid (MP) clade of Fabaceae. Likewise, de la Estrella et al. (2017) explored the evolutionary origins of the Detarioideae clade within Fabaceae, emphasizing its post-Gondwanan diversification and terra firme adaptation as a key factor in the early evolution of this legume lineage. Oyebanji et al. (2021) demonstrated that climate change will likely shift the spatial distribution of endemic legume species in the Guineo-Congolian (GC) region, leading to a decline in their future distribution and potentially threatening their persistence. Du Preez et al. (2025) identified unique global biogeographic patterns in *Indigofera* L., proposing that the tribe *Indigofereae* originated in the Africa-Madagascar region, with *Indigofera* itself tracing its evolutionary origins to mainland Africa approximately 38 million years ago. Additionally, Yang et al. (2022) found that Quaternary climate change and environmental energy were key drivers of diversity patterns in the Mediterranean genus *Medicago* L., with their effects remaining consistent across global, continental, and biome scales. Leveraging the global databases of plant diversity and distribution, the present study offers a macroecological assessment of the patterns and drivers of species and genera richness of Fabaceae across the globe. More specifically, the present study aimed to answer the following research questions:

- What are the global patterns in the species and genera richness of Fabaceae?
- Does the species and genera richness of Fabaceae exhibit a latitudinal diversity gradient?
- How does species richness pattern of Fabaceae vary across continents and biomes?
- What are the potential climatic, environmental, and topographical drivers that shape the global patterns of species and genera richness of Fabaceae across globe?

By addressing these questions, the present will shed light on the distribution patterns of the Fabaceae family and deepen our understanding of the climatic and environmental drivers that influence its diversity patterns around the world.

## Materials and methods

### Diversity and distribution data

The global taxonomic and distribution data of Fabaceae was retrieved from the *World Checklist of Vascular Plants* (WCVP) (Govaerts et al., 2021; Brown et al., 2023) (http://wcvp.science.kew.org/) and *Global Inventory of Floras and Traits* (GIFT) (Weigelt et al., 2020; Denelle et al., 2023) (https://gift.uni-goettingen.de/), accessed on 13 January 2025. The WCVP is the most extensive and carefully maintained database of vascular plants, offering details on each species’ growth form, distribution, and biome affiliation according to the Level-3 units of the World Geographical Scheme for Recording Plant Distributions (WGSRPD) (i.e., 369 botanical countries) (Brummitt et al., 2001). Likewise, the GIFT database contains regional plant inventories that have been generated from checklists and published floras for 3,400 different geographic regions worldwide. These regions include islands, protected areas, biogeographical regions (like botanical countries), and political units (like provinces and countries) (Weigelt et al., 2020). I extracted the native diversity and distribution data of Fabaceae at two biogeographical levels of botanical countries (hereafter countries) and botanical continents (hereafter continents) worldwide using the WGSRPD classification. Recent studies on plant biogeography and macroecology have used a similar biogeographical classification to investigate the global patterns of species diversity (Sandel et al., 2020; Cai et al., 2023; Qian et al., 2024). Fabaceae species designated as extinct, doubtful, or introduced by the WCVPfor a particular country were not included. At the continental level, if a species was recorded as introduced in certain countries within the continent, it was included in the analysis for the entire continent. Further, the taxonomic information of the Fabaceae species from the GIFT is based on the WCVP, therefore, I directly combined the distribution data retrieved from GIFT with that extracted from WCVP (Cai et al., 2023). The Fabaceae checklists retrieved from GIFT for smaller regions were consolidated with their respective countries to align with the World Geographical Scheme for Recording Plant Distributions (WGSRPD) classification. If species lists from both sources were available for a region, they were merged to create a unique set of species, ensuring no duplicates. The integration of data from the World Checklist of Vascular Plants (WCVP) and the Global Inventory of Floras and Traits (GIFT) has been widely adopted in recent biodiversity research studies to investigate large-scale spatial patterns of plant diversity (see Cai et al., 2023; Qian and Qian, 2023; Taylor et al., 2023). The final dataset included a total of 27,421 taxa of Fabaceae belonging to 794 genera distributed in 351 countries and 8 continents (Asia-Tropical, Asia-Temperate, Australasia, Europe, Northern America, Southern America, Africa, and Pacific) worldwide.

### Predictors of species and genera richness

Generally, several ecological and biogeographical processes are expected to influence species richness (SR), across the world (Kier et al., 2009; Cai et al., 2023; Tordoni et al., 2024). Following this expectation, a collection of explanatory factors that characterize these processes were identified and divided them into four categories: long-term climatic stability (LTCS), current climate (CUCL), environmental heterogeneity (ENHE), and geography (GEOG). For the latter two groups, the selected variables include: geographical area (Area)(km^2^) and elevational range (ELR) (m), topographic position index (TPI), and terrain ruggedness index (TRI), obtained from EarthEvn (https://www.earthenv.org/; Amatulli et al., 2018). The elevation range of each country was calculated as the difference between the maximum and minimum elevation. The current climatic variables selected included a total of 19 variables (for more details, see Fick and Hijmans, 2017; https://www.worldclim.org/)(**Appendix Table S1**). The long-term climatic stability variables selected include: temperature stability (Tstab) (°C) and precipitation stability (Pstab) (mm), temperature anomaly (Tanom) (°C), precipitation anomaly (Panom) (mm), and velocity of temperature change (Tvel) since the last glacial maximum (LGM).To calculate the Tstab and Pstab for each country, I utilized the R package “climateStablity” version 0.1.4 (Owens and Guralnick, 2019). The variables like Tanom and Panom since the LGM were calculated as the differences in mean annual temperature and annual precipitation between the LGM and the present, respectively (Sandel et al., 2020; Cai et al., 2023). These explanatory variables have been widely used for studying the regional and global patterns of biodiversity across various temporal and spatial scales (Kier et al., 2009; Sandel et al., 2020; Cai et al, 2023; Qian et al., 2024). The climatic variables were obtained from WorldClim version 2.1 (Fick and Hijmans, 2017; https://www.worldclim.org/) and PaleoClim (Brown et al., 2018; http://www.paleoclim.org/) at a resolution of 30-arc-second (**Appendix Table S1**). I calculated the mean of each climatic variable for each country of the world for the downstream analysis. Before analysis, I used Pearson’s correlation at the threshold of 0.75 to check the multicollinearity among the 28 predictors (**Appendix Figure S1**). After the multicollinearity test, I selected a total of 12 variables out of 28 variables to estimate their role in explaining the variation of species and genera richness of Fabaceae across the world. The final set of predictors included: mean annual temperature (Tmean) (°C), minimum temperature of the coldest month (Tmin) (°C), temperature seasonality (Tseas) (°C), temperature diurnal range (Tdur) (°C), annual precipitation (Pmean) (mm),precipitation seasonality (Pseas) (mm), geographical area (km^2^), elevation range (ELR) (m), topographic position index (TPI), and terrain ruggedness index (TRI), precipitation anomaly (Panom) (mm), and temperature stability (Tstab) (°C).

### Data analyses

All data analyses were performed in R version 4.1.2 (R Core Team, 2022).The chi-square test was used to investigate whether there are significant differences in the observed numbers of Fabaceae species among the continents in terms of representation of species with different biome affiliations in “vcd” package version 0.1.7 (Meyer et al., 2020). A generalized linear model with a negative binomial approach was performed to investigate the relative role of different selected explanatory variables in shaping the global patterns of species and genera richness of Fabaceae. Before performing the negative binomial approach, *dispersiontest()* function of the “AER” package version1.2.6 (Kleiber and Zeileis, 2008) was used to check the overdispersion in the response variables (species and genera richness). I used *glm.nb()* function in the “MASS” package version 7.3.5 (Venables and Ripley, 2002) to perform the negative binomial regression. Initially, I started with a full model that included all of the selected explanatory variables, and then new models were generated by using all possible combinations of explanatory variables. I used the corrected Akaike Information Criterion (AICc) for the model selection, and the model with the lowest AICc value is considered as the best model (Arnold, 2010). For different model comparisons, I used *dredge()* function from the “MuMIn” package version 1.43.17 (Barton, 2020). Furthermore, hierarchical partitioning analysis was performed in the “glmm.hp” package version 0.1.0 (Lai et al., 2022) to assess the individual contributions of each selected variable to marginal R^2^ in explaining variance in species and genus richness of Fabaceae across the globe.

## Results

### Global patterns of species richness of Fabaceae

The species richness of Fabaceae varied greatly among the countries of the world. The maximum number of species was found in tropical regions of the world. Western Australia harbors a maximum number of species (1825) located in Australasia, followed by Brazil North (1643 species), Brazil Southeast (1545 species), Zaire (1506 species), Mexico Southwest (1496 species), Iran (1475 species), Tanzania (1455 species), Colombia (1445 species), Brazil West-Central (1430 species), and Cape Provinces (1426 species). On an average basis, each species was distributed among ~ 4countries across the globe. Seven species (*Canavalia rosea* (Sw.) DC., *Rhynchosia minima* (L.) DC., *Guilandina bonduc* L., *Grona triflora* (L.) H.Ohashi & K.Ohashi, *Vicia sativa* L., *Vigna luteola* (Jacq.) Benth. was found to be distributed in more than 100countries.

### Global patterns of genera richness of Fabaceae

**Figure 1:**
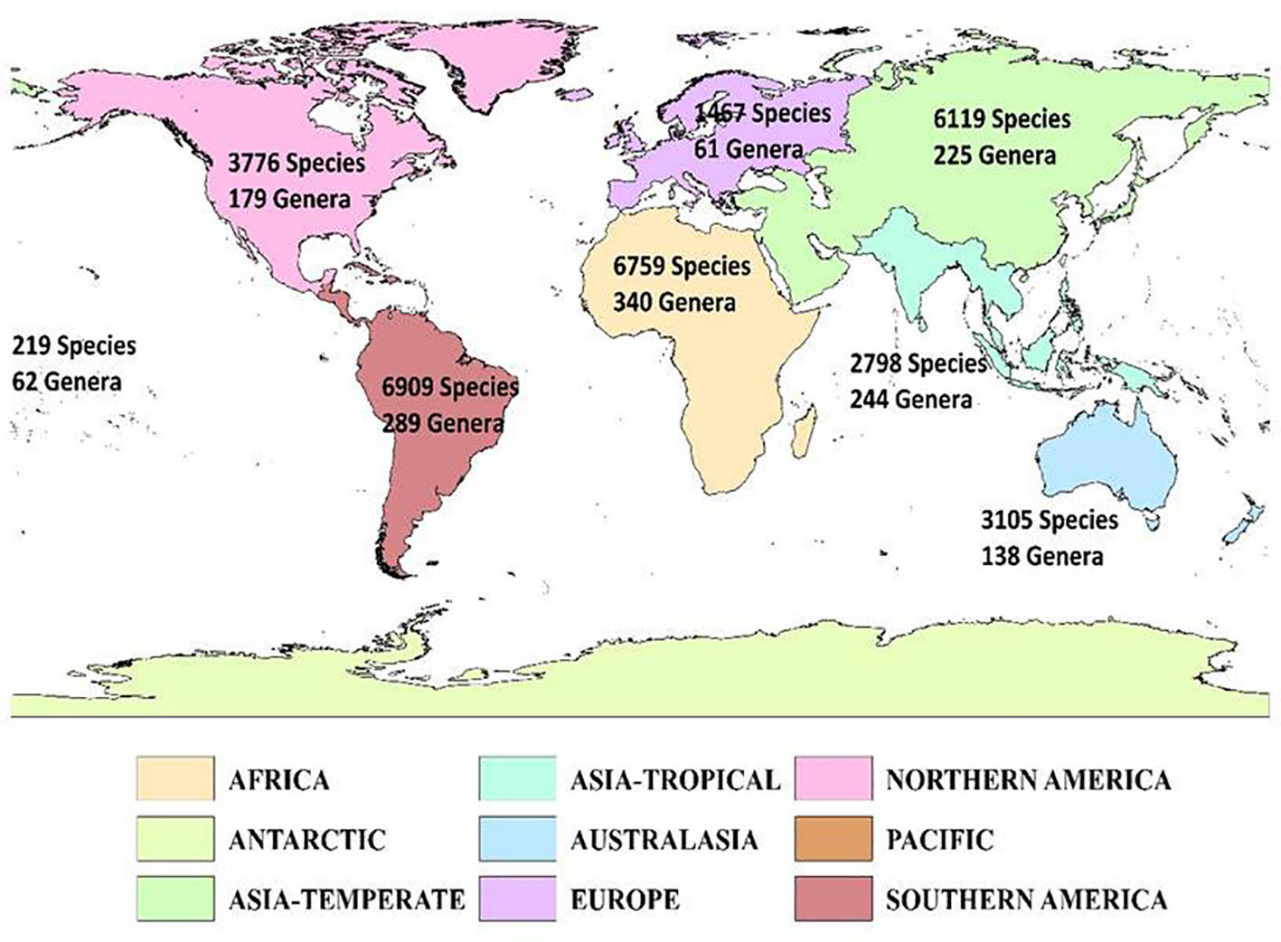
Geographic patterns of genera richness of Fabaceae among the countries across the globe. Islands are represented by circles, with size reflecting the number of genera. The map was generated using the Mollweide projection.

The genera richness of Fabaceae showed a considerable variation among the countries of the world, with Bolivia harboring maximum number(174 genera), Zaire (172 genera), Brazil North (166 genera), Colombia (165 genera), Brazil Northeast (163 genera), Cameroon (163 genera), Tanzania (156 genera), Venezuela (155 genera), Brazil Southeast (151 genera) and Brazil West-Central (148 genera) (**Figure 1**). Likewise, each genus of Fabaceae was distributed among 22countries on an average, with *Astragalus* L. in 206 countries, *Crotalaria* L. (198 countries), *Vicia* L. (194 countries), *Lathyrus* L. (188 countries), *Tephrosia* Pers. (187 countries), *Senna* Mill. (186 countries), *Trifolium* Tourn. ex L. (183 countries), *Canavalia DC*. (175 countries), *Indigofera* L. (175 countries), and *Chamaecrista* (L.) Moench in 173 countries. I also found 94 genera (11.83%) that were restricted to a single country across the globe.

### Latitudinal patterns of species and genera richness of Fabaceae

**Figure 2:**
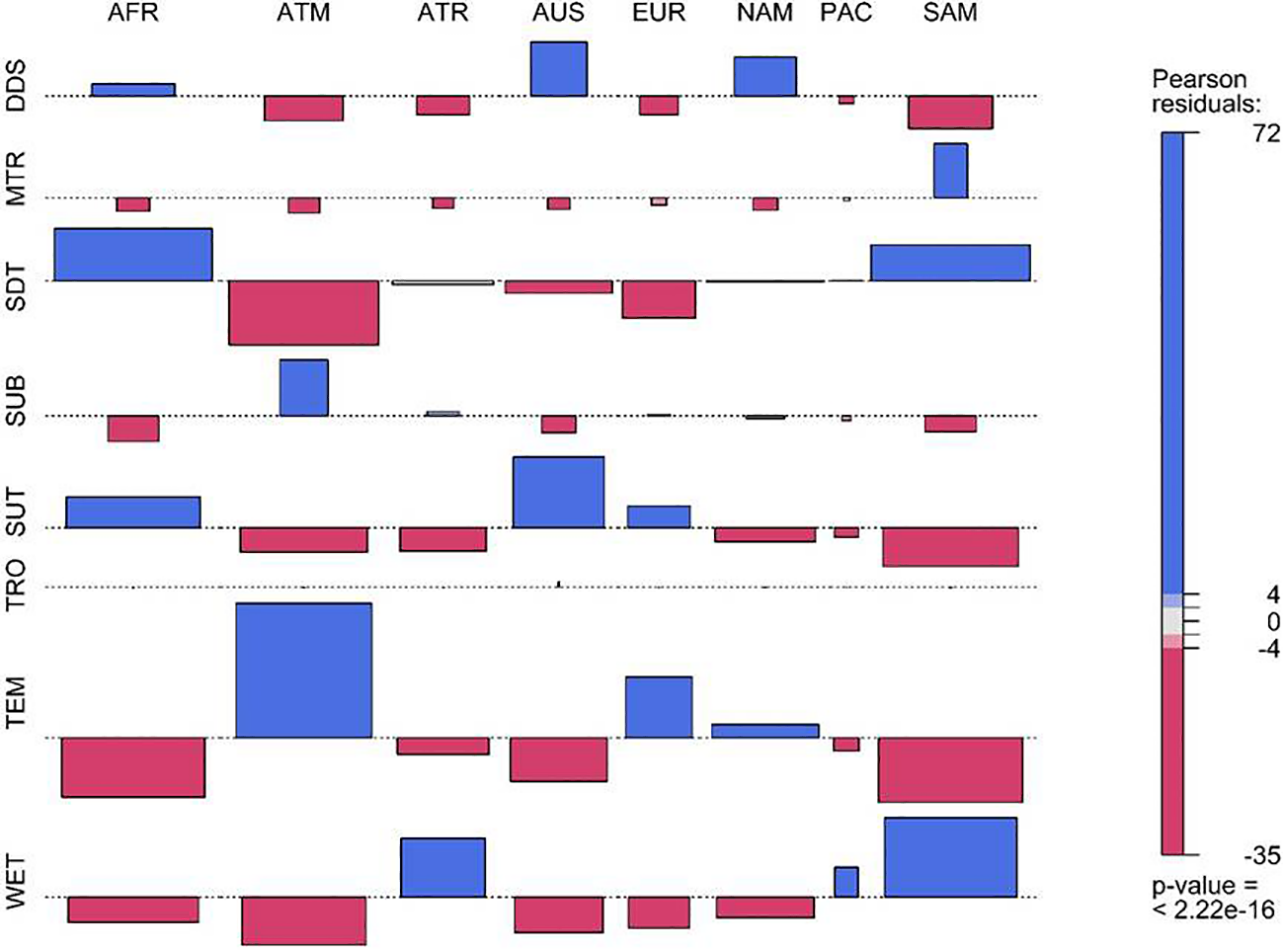
Relationship of **(a)** species and **(b)** genera richness of Fabaceae with the latitude.

The generalized additive model revealed a significant negative relationship of species and genera richness of Fabaceae with the latitude (**Figure 2**), thereby supporting the latitudinal diversity gradient, which posits that biodiversity generally decreases from the equator toward the poles.

### Continental patterns of Fabaceae

**Figure 3:**
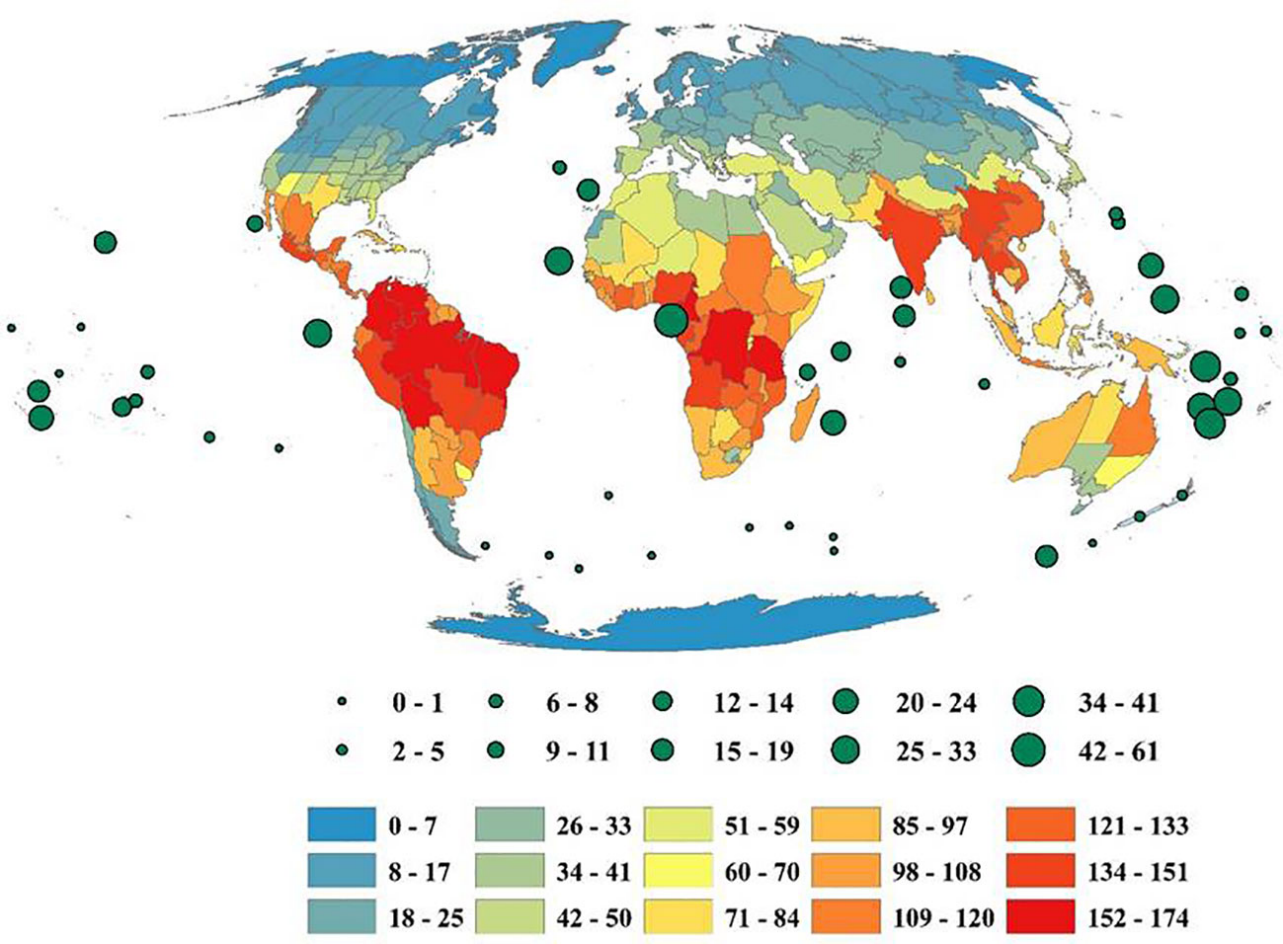
Geographic patterns of species and genera richness of Fabaceae among the continents of the globe.

The results revealed a substantial variation in the number of species and genera of Fabaceae among the eight continents across the world (**Figure 3**). Southern America harbors the highest number of 6909 species, followed by Africa (6759 species), Asia-Temperate (6119 species), Northern America (3776 species), Australasia (3105 species), Asia-Tropical (2798 species), Europe (1467 species) and Pacific (219 species) (**Figure 3**). 6053 species were found restricted to Southern America only. There are 5902 species that are restricted to Africa, 4362 to Asia-Temperate, 2902 species to Northern America, 2882 to Australasia, 1617 species to Asia-Tropical, 628 to Europe, and 127 species to the Pacific. In contrast, Africa harbors the highest number of genera (340), followed by Southern America (289 genera), Asia-Tropical (244 genera), Asia-Temperate (225 genera), Northern America (179 genera), Australasia (138 genera), Pacific (62 genera) and Europe (61 genera) (**Figure 3**). Additionally, 173 genera of Fabaceae were found unique to Africa, 139 genera to Southern America, 50 genera to Australasia, 32 genera to Asia-Tropical, 29 genera to Northern America and, 13 genera distributed in Asia-Temperate only.

### Biome affiliation

**Figure 4:**
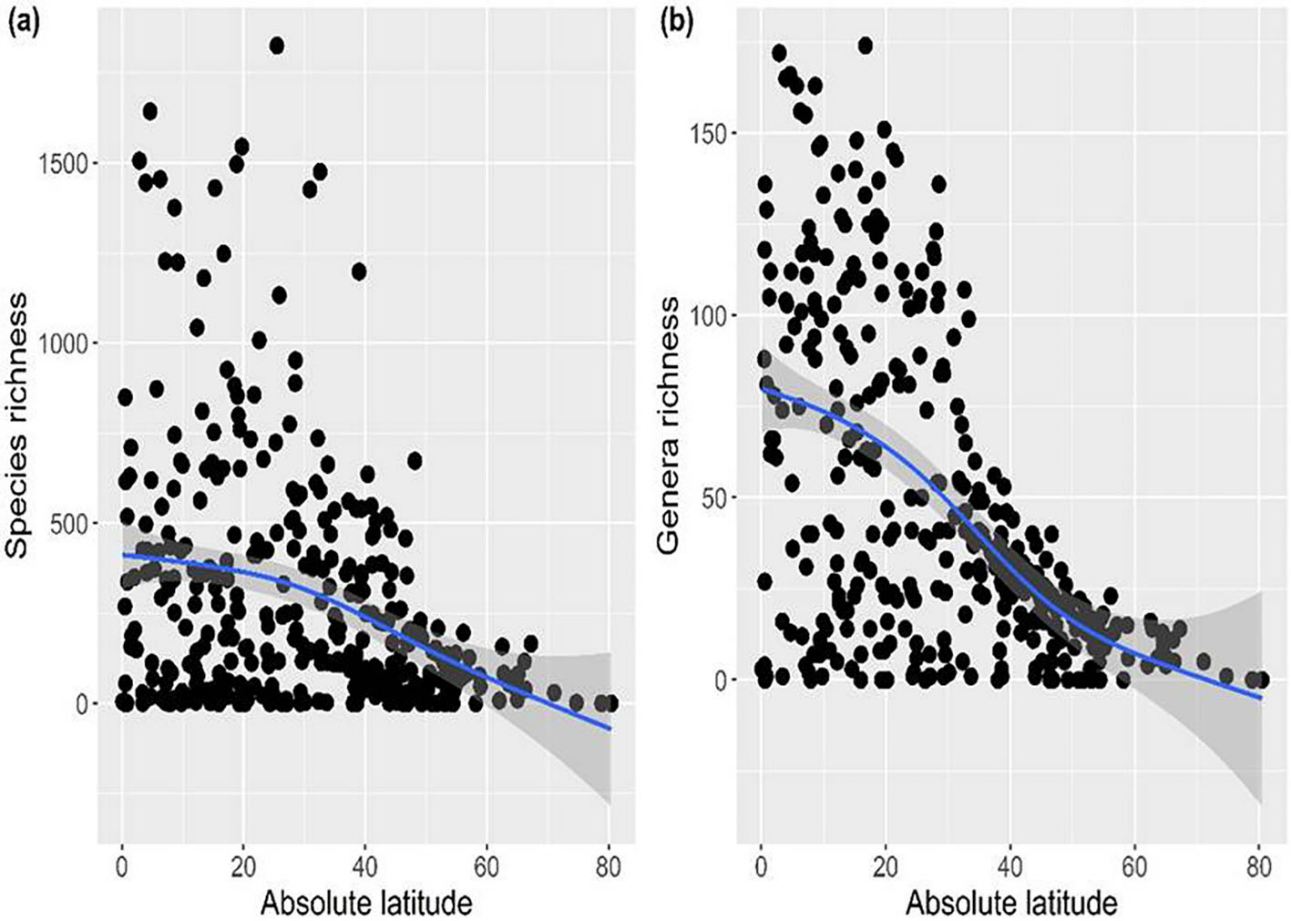
Mosaic plot showing the comparison of observed and expected numbers of Fabaceae species **(a)** and genera **(b)** according to their biome affiliation in different countries of the world. The corresponding Pearson’s residuals, which were obtained from the contingency table, are shown by color. Positive residual values indicate greater observed values than predicted, whilst negative residual values indicate lower observed values than expected. [DDS, desert or dry shrubland; MTR, montane tropical; SDT, seasonally dry tropical; SUB, subalpine or subarctic; SUT, subtropical; TRO, tropical; TEM, temperate; WET, wet tropical; AFR, Africa; ATM, Asis-Temperate; ATR, Asia-Tropical; AUS, Australasia; EUR, Europe; NAM, Northern America; PAC, Pacific; and SAM, Southern America].

The results revealed that the majority of the species of Fabaceae are affiliated to seasonally dry tropical biome (7562 species; 27.57%), followed by temperate biome (6051 species; 22.06%), subtropical biome (5402; 19.70%), wet tropical biome (5102; 18.60%), desert or dry shrubland biome (2145 species; 7.83%), subalpine or subarctic biome (793; 2.90%), and montane tropical biome (366 species; 1.34%). Based on the Chi-square test, it was found that the distribution of Fabaceae species belonging to different biomes varied significantly among the continents of the world (*χ^2^ =* 23864; *df* = 49; *p*< 0.01) (**Figures 4a, b**). The species belonged to desert or dry shrubland (DDS) are over-represented in the Africa, Australasia and Northern America. Likewise, the species affiliated to the montane tropical biome (MTR) are highly represented in Southern America and under-represented in the rest of the countries (**Figures 4a, b**). I also found seasonally dry tropical (SDT) species over-represented in Africa and Southern America and under-represented in Asia-Temperate, Australasia, and Europe. Similarly, species affiliated with the temperate biome (TEM) were over-represented in Asia-Temperate, Europe, and Northern America (**Figure 2**). Furthermore, a higher representation of species was found belonging to the wet tropical biome (WET) in Asia-Tropical and Southern America (**Figures 4a, b**).

### Drivers of species and genera richness

**Figure 5:**
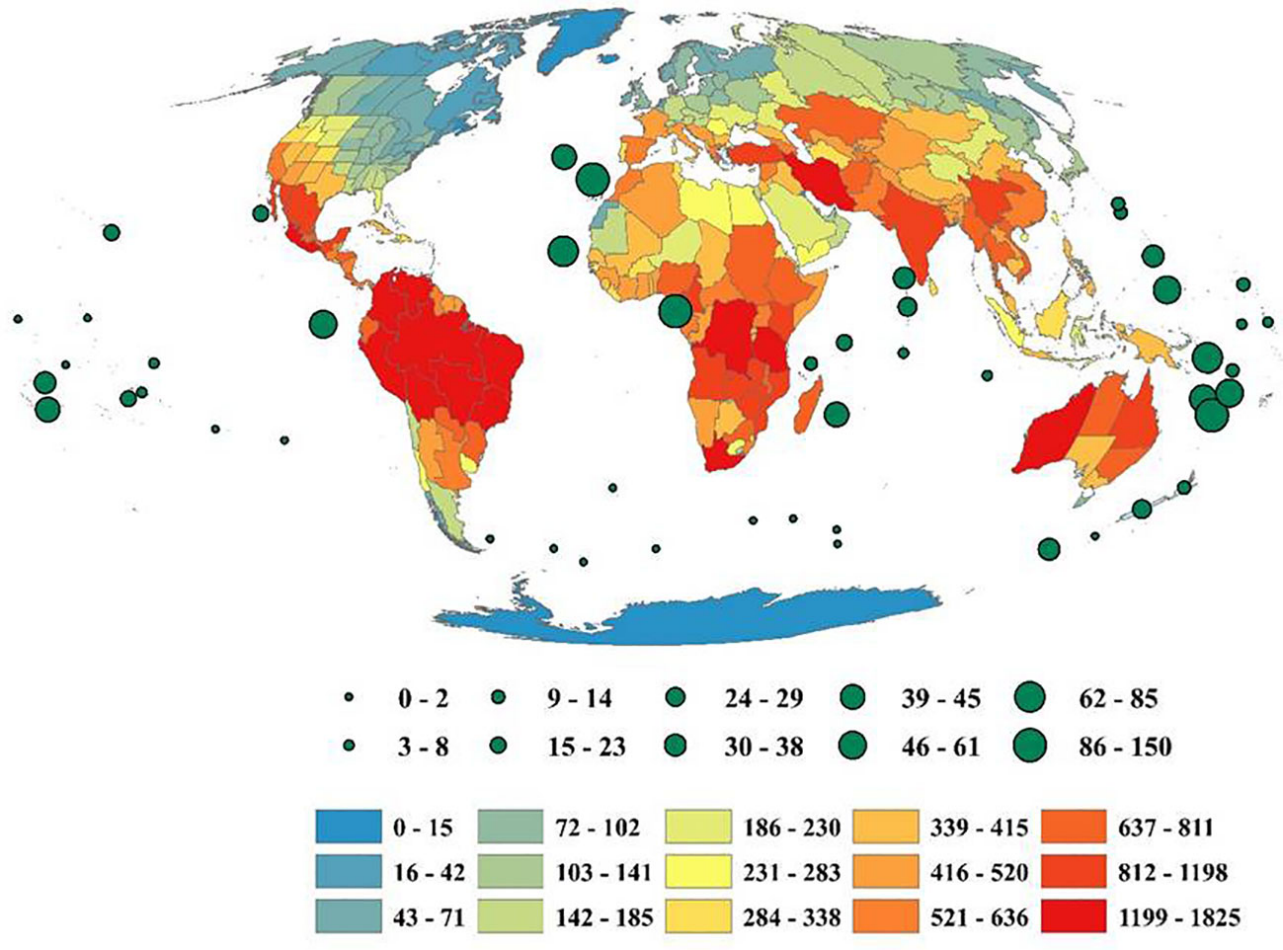
Standardized coefficient of the negative binomial model for **(a)** species richness and **(b)** genera richness of Fabaceae. Significant variables are shown as ***p < 0.001, **p < 0.01, and *p < 0.05. The red lines represent a negative effect, while as blue lines indicate a positive effect of variables. [Tmean, mean annual temperature; Tmin, minimum temperature of the coldest month; Tseas, temperature seasonality; Tdur, temperature diurnal range; Pmean, annual precipitation; Pseas, precipitation seasonality, area; ELR, elevation range; TPI, topographic position index; TRI, terrain ruggedness index; Panom, precipitation anomaly; Tstab, temperature stability].

Based on the negative binomial model, a set of explanatory variables responsible for shaping the patterns of species and genera richness of Fabaceae were identified among the countries of the world. For each of the response variables (species and genera richness), a total of 4095 models were developed based on possible combinations of the 12 explanatory variables. In the case of species richness, the best model was found as the combination of ELR + Pseas + Tdur + Tmean + Area (AICc = 4538.83; AICc weight = 0.039). All the significant variables showed a positive effect on the species richness of Fabaceae (**Figure 5**). The model including all the selected explanatory variables explained about 62% of the variation in the species richness. The results of hierarchical partitioning analysis revealed that elevation range (ELR) explained the maximum amount of variation (28.88%) in species richness, followed by temperature diurnal range (Tdur) (24.56%).

Different sets of predictors were found to be responsible for explaining the variation of genera richness of Fabaceae among the countries of the world (**Figure 5**). Out of 4095 models, the best model was found as the combination of ELR + Pseas + Tdur + Tmean + Area + Pmean + Tstab+Tseas (AICc = 3211.25; AICc weight = 0.0625). All the significant predictors except temperature seasonality (Tseas) showed a positive effect on the genera richness of Fabaceae. About 67% of variation was explained in genera richness by including all the selected variables. Additionally, temperature diurnal range (Tdur) was found as the significant predictor in explaining the maximum variation in genera richness(20.24%), followed by temperature seasonality (Tseas) (16.76%) and elevation range (ELR) (14.48%).

## Discussion

The present study offers a thorough macroecological evaluation of the Fabaceae family on a global scale by analyzing its distribution patterns and the underlying environmental and climatic drivers. Despite the ecological and economic importance of Fabaceae, global-scale studies on its species and genera richness remain scarce, and previous research has largely lacked an explicit geographical framework. Leveraging recent advancements in data integration, analytical and methodological approaches, investigating the patterns and drivers of this highly diverse and economically important family enhances our understanding of plant species and genera richness and contributes to evidence-based conservation strategies.

The findings show that the Fabaceae species and genus richness vary across tropical and temperate zones, with maximum richness centers in tropical regions. This geographic pattern in species and genera is consistent with the latitudinal diversity gradient, which states that species richness declines as latitude increases. The observed variation in species and genera richness along latitudinal gradients is consistent with previous global studies (Lavin et al., 2004; Kreft and Jetz, 2007; Sabatini et al., 2022; Cai et al., 2023; Oyebanji et al., 2023). Notably, the observed richness centers overlap with recently mapped global locations of critical natural assets essential for ecosystem services (Chaplin-Kramer et al., 2023). The increased species and genus richness of Fabaceae in tropical regions can be linked to its historical biogeographic origin and sustained radiation in these habitats (Zhao et al., 2021; Oyebanji et al., 2023). This finding supports the tropical conservatism hypothesis, which posits that most angiosperm lineages, including Fabaceae, originated and diversified during the Cenozoic, primarily in stable tropical environments (Peterson et al., 1999; Wiens et al., 2010). The dominance of Fabaceae species in seasonally dry tropical biomes can be attributed to their remarkable ecological adaptations, including nitrogen fixation, drought tolerance, and efficient reproductive strategies (Oyebanji et al., 2023). A combination of physiological, evolutionary, and environmental reasons contributes to the dominance of Fabaceae in tropical climates (Lavin et al., 2005; Oyebanji et al., 2023). By forming symbiotic relationships with *Rhizobium* bacteria, Fabaceae can fix nitrogen biologically, which gives them a competitive edge over other plant families and allows them to colonize nutrient-deficient soils (Lavin et al., 2005; Oyebanji et al., 2023). Furthermore, speciation in Fabaceae might be encouraged by high rates of evolution, niche specialization, and adaptive radiation across a variety of environments, including but not limited to tropical regions (Lavin et al., 2004). Taken together, these interrelated processes may support the remarkable species diversity and ecological dominance of the Fabaceae in tropical regions.

The findings of the present study reveal a considerable variation in the distribution of Fabaceae species across different biomes, with the highest species richness observed in the SDT, followed by the TEM and SUT.A statistically significant variation in the biome-wise distribution of Fabaceae species was found across continents, indicating that biogeographical and climatic factors shape the global distribution of this family. The ability of Fabaceae to fix nitrogen, withstand drought, and reproduce effectively accounts for their dominance in seasonally dry tropical biomes (Oyebanji et al., 2023). In many Acacia species, the presence of reduced leaves and phyllodes, which serve as the main photosynthetic structures, reflects a suite of adaptations to arid environments (Renner et al., 2021). These are complemented by deep root systems and drought-resilient features such as compound leaves, allowing them to cope effectively with prolonged water scarcity (de la Estrella et al., 2017). Because of these adaptations, Fabaceae species may survive and spread throughout seasonally dry tropical biomes, which are common in South America and Africa and where SDT species are overrepresented. These findings are consistent with the previous study showing that Fabaceae species are common in tropical deciduous forests and savannas, which suffer from seasonal drought and depend on adaptations like deep-rooting systems and drought-resistant leaves (Oyebanji et al., 2023). The overrepresentation of Fabaceae species in tropical and subtropical regions concords with the tropical conservatism hypothesis, which posits that most angiosperm lineages, including Fabaceae, originated in warm tropical climates and subsequently radiated into other biomes (Peterson et al., 1999; Wiens et al., 2010). Understanding the factors that contribute to uneven species richness and composition between tropical seasonal and moist (with and without a pronounced dry season, respectively) is critical for predicting potential species extinction due to drought-induced mortality (Cássia-Silva et al., 2019). Fabaceae species that belong to the desert or dry shrubland (DDS) biome are overrepresented in North America, Africa, and Australasia. This is mainly because of their xeromorphic traits, which minimize water loss, thickened cuticles, and specialized seed dispersal mechanisms (Arakaki et al., 2011). The Fabaceae family is highly represented in the wet tropical biome (WET) in Asia-Tropical and South America. Adaptations like fast growth rates, tolerance to shade, and mutualistic relationships with pollinators and mycorrhizal fungi allow these plants to persist in high-humidity, high-rainfall conditions (Lavin et al., 2004; Lewis et al., 2005). The uneven representation of Fabaceae species across biomes warrants further investigation in future studies, particularly through the lens of biome-specific historical and climatic factors such as the timing of aridification, glaciation events, and long-term climate stability to better understand biome-level distribution patterns.

The findings revealed that multiple climatic and geographic factors shape the species and genera richness patterns of Fabaceae across countries, with key predictors differing between taxonomic levels. Species richness was primarily influenced by elevation range (ELR), temperature diurnal range (Tdur), precipitation seasonality (Pseas), annual mean temperature (Tmean), and geographical area (area). These factors align with previous findings emphasizing the importance of topographic heterogeneity and climatic variability in promoting species diversity (Kreft and Jetz, 2007; Stein et al., 2014; Song et al., 2024). The strong influence of ELR suggests that greater altitudinal variation fosters diverse microhabitats, enabling species accumulation and niche differentiation (Rahbek, 2005; Stein et al., 2014). Our findings concord with Folk et al. (2024), who demonstrated that environmental heterogeneity plays a crucial role in driving the diversification of *Astragalus*, one of the most species-rich genera within Fabaceae. Additionally, the diurnal temperature range, which represents daily thermal variations, may affect transpiration and photosynthesis in plants, which in turn may affect the distribution of species (Zhao et al., 2021). On the other hand, a wider range of environmental factors, such as ELR, Tdur, Pseas, Tmean, area, annual mean precipitation (Pmean), temperature stability (Tstab), and temperature seasonality (Tseas), influenced genus richness. Adding more climate factors implies that genera richness reacts to wider climatic gradients, which could have long-term effects on lineage persistence and evolutionary diversity. Because seasonal variations in precipitation can affect plant reproductive cycles and habitat appropriateness, the effect of precipitation-related parameters (Pseas and Pmean) highlights the importance of water availability in sustaining the diversity of Fabaceae across globe (Stein et al., 2014; Cai et al., 2023; Tordoni et al., 2024). Furthermore, temperature seasonality (Tseas) and temperature stability (Tstab) provide stable circumstances that support long-term species survival and diversification, they further emphasize the significance of climatic constancy in preserving genus-level diversity (Cai et al., 2023). The stability in temperature and seasonality likely facilitated the steady diversification of lineages within genera, potentially driven by higher speciation rates, lower extinction and turnover rates, or a combination of these factors. Nonetheless, further studies are needed to disentangle these processes and fully understand their roles in shaping current diversity patterns of Fabaceae. The results support the ecological theory that environmental heterogeneity promotes biodiversity by generating a variety of appropriate habitats (Rahbek, 2005; Stein et al., 2014). The spatial patterns of Fabaceae richness across various climatic zones are ultimately shaped by these eco-physiological reactions and various ecological factors (Lavin et al., 2005; Oyebanji et al., 2023; Folk et al., 2024). Overall, the present study sheds light on the main environmental factors that determine the diversity of Fabaceae and how these factors vary depending on the taxonomic level. Comprehending the interactions among the climatic and environmental variables is essential for conserving biodiversity, especially in light of climate change, which may modify the patterns of precipitation and temperature, thereby affecting the distribution of species.

## Conclusions and conservation implications

The present study offers a global macroecological assessment of the Fabaceae family by investigating the species and genus richness patterns as well as the underlying climatic and environmental drivers. Based on the various data-driven approaches, it was emphasized how climate and environmental heterogeneity influences the global distribution of this commercially and ecologically important plant family. The global distribution patterns of the Fabaceae concords with the well-established biodiversity gradients, including the latitudinal diversity gradient and the tropical conservatism hypothesis. The ecological dominance of Fabaceae in tropical regions is attributed to its high evolutionary adaptability, physiological resilience, and symbiotic nitrogen fixation, which enable its diversification and persistence. Moreover, this study underscores the importance of macroecological drivers in determining species and genera richness across countries. The findings of the present study will aid in understanding the global biodiversity patterns and give empirical support for ecological theories such as the energy limitations hypothesis and the historical perturbation hypothesis. The findings also identify important hotspots for Fabaceae where species are overrepresented, especially in seasonally dry tropical biomes, which are among the most threatened ecosystems due to deforestation and climate change (Siyum, 2020). Given the ecological significance of the family in soil stabilization, nitrogen cycling, and food security, our results highlight the necessity of targeted conservation initiatives in these vulnerable areas. Understanding the biome-specific adaptations of Fabaceae can also aid in ecological restoration operations and long-term land use planning. Looking forward, the results of this study will offer important insights that support achieving the diverse objectives outlined in the Global Strategy for Plant Conservation and the Post-2020 Global Biodiversity Framework of the Convention on Biological Diversity.

## Data Availability

The original contributions presented in the study are included in the article/**Supplementary Material**. Further inquiries can be directed to the corresponding author/s.

## References

[B1] Amatulli G. Domisch S. Tuanmu M. N. Parmentier B. Ranipeta A. Malczyk J. . (2018). A suite of global, cross-scale topographic variables for environmental and biodiversity modeling. Sci. Data 5, 1–15. doi: 10.1038/sdata.2018.40, PMID: 29557978 PMC5859920

[B2] Arakaki M. Christin P. A. Nyffeler R. Lendel A. Eggli U. Ogburn R. M. . (2011). Contemporaneous and recent radiations of the world’s major succulent plant lineages. Proc. Natl. Acad. Sci. 108, 8379–8384. doi: 10.1073/pnas.1100628108, PMID: 21536881 PMC3100969

[B2000] Arnold T. W. (2010). Uninformative parameters and model selection using Akaike's Information Criterion. J. Wildl. Manage. 74 (6), 1175–1178. doi: 10.1111/j.1937-2817.2010.tb01236.x, PMID: 41808597

[B3] Barton K. (2020). MuMIn: Multi-Model Inference. R package version 1.43.17. Available online at: https://CRAN.R-project.org/package=MuMIn (Accessed February 16, 2025).

[B4] Baselga A. (2010). Partitioning the turnover and nestedness components of beta diversity. Global Ecol. biogeography 19, 134–143. doi: 10.1111/j.1466-8238.2009.00490.x, PMID: 41808597

[B5] Benton M. J. Wilf P. Sauquet H. (2022). The Angiosperm Terrestrial Revolution and the origins of modern biodiversity. New Phytol. 233, 2017–2035. doi: 10.1111/nph.v233.5, PMID: 34699613

[B6] Brown J. L. Hill D. J. Dolan A. M. Carnaval A. C. Haywood A. M. (2018). PaleoClim, high spatial resolution paleoclimate surfaces for global land areas. Sci. Data 5, 1–9. doi: 10.1038/sdata.2018.254, PMID: 30422125 PMC6233254

[B7] Brown M. J. Walker B. E. Black N. Govaerts R. H. Ondo I. Turner R. . (2023). rWCVP: a companion R package for the World Checklist of Vascular Plants. New Phytol. 240, 1355–1365. doi: 10.1111/nph.v240.4, PMID: 37289204

[B8] Brummitt R. K. Pando F. Hollis S. Brummitt N. A. (2001). World geographical scheme for recording plant distributions Vol. 951 (Geneva, Switzerland: International working group on taxonomic databases for plant sciences (TDWG), 952.

[B9] Cadotte M. W. Carscadden K. Mirotchnick N. (2011). Beyond species: functional diversity and the maintenance of ecological processes and services. J. Appl. Ecol. 48, 1079–1087. doi: 10.1111/j.1365-2664.2011.02048.x, PMID:

[B10] Cai L. Kreft H. Denelle P. Taylor A. Craven D. Dawson W. . (2024). Environmental filtering, not dispersal history, explains global patterns of phylogenetic turnover in seed plants at deep evolutionary timescales. Nat. Ecol. Evol., 1–11. doi: 10.1038/s41559-024-02599-y, PMID: 39613945

[B11] Cai L. Kreft H. Taylor A. Denelle P. Schrader J. Essl F. . (2023). Global models and predictions of plant diversity based on advanced machine learning techniques. New Phytol. 237, 1432–1445. doi: 10.1111/nph.v237.4, PMID: 36375492

[B12] Cássia-Silva C. Freitas C. G. Alves D. M. Bacon C. D. Collevatti R. G. (2019). Niche conservatism drives a global discrepancy in palm species richness between seasonally dry and moist habitats. Global Ecol. Biogeography 28, 814–825. doi: 10.1111/geb.12895, PMID: 41808597

[B10000] Chaplin-Kramer R. Neugarten R. A. Sharp R. P. Collins P. M. Polasky S. Hole D. . (2023). Mapping the planet's critical natural assets. Nat. Ecol. Evol. 7(1), 51–61. 36443466 10.1038/s41559-022-01934-5 PMC9834042

[B14] Chartier M. von Balthazar M. Sontag S. Löfstrand S. Palme T. Jabbour F. . (2021). Global patterns and a latitudinal gradient of flower disparity: perspectives from the angiosperm order Ericales. New Phytol. 230, 821–831. doi: 10.1111/nph.v230.2, PMID: 33454991 PMC8048689

[B15] Crist T. O. Veech J. A. Gering J. C. Summerville K. S. (2003). Partitioning species diversity across landscapes and regions: a hierarchical analysis of α, β, and γ diversity. Am. Nat. 162, 734–743. doi: 10.1086/378901, PMID: 14737711

[B10001] de la Estrella M. Forest F. Wieringa J. J. Fougère-Danezan M. Bruneau A (2017). Insights on the evolutionary origin of Detarioideae, a clade of ecologically dominant tropical African trees. New Phytolog. 214 (4), 1722–1735. 10.1111/nph.14523 28323330

[B17] Denelle P. Weigelt P. Kreft H. (2023). GIFT—An R package to access the Global Inventory of Floras and Traits. Methods Ecol. Evol. 14, 2738–2748. doi: 10.1111/2041-210X.14213, PMID: 41808597

[B18] Diniz Filho J. A. F. Jardim L. Guedes J. J. Meyer L. Stropp J. Frateles L. E. F. . (2023). Macroecological links between the Linnean, Wallacean, and Darwinian shortfalls. Front. Biogeography 15.

[B19] Du Preez B. Schrire B. D. Dreyer L. L. Stirton C. H. Chimphango S. B. Muasya A. M. (2025). Global biogeographic patterns of the genus Indigofera (Fabaceae: Indigofereae). Braz. J. Bot. 48, 19. doi: 10.1007/s40415-024-01045-4, PMID:

[B21] Fick S. E. Hijmans R. J. (2017). WorldClim 2: new 1-km spatial resolution climate surfaces for global land areas. Int. J. climatology 37, 4302–4315. doi: 10.1002/joc.2017.37.issue-12, PMID: 41810896

[B22] Folk R. A. Charboneau J. L. Belitz M. Singh T. Kates H. R. Soltis D. E. . (2024). Anatomy of a mega-radiation: Biogeography and niche evolution in Astragalus. Am. J. Bot. 111, e16299. doi: 10.1002/ajb2.v111.3, PMID: 38419145

[B23] Friedman J. (2020). The evolution of annual and perennial plant life histories: ecological correlates and genetic mechanisms. Annu. Rev. Ecology Evolution Systematics 51, 461–481. doi: 10.1146/annurev-ecolsys-110218-024638, PMID: 41139587

[B24] Govaerts R. Nic Lughadha E. Black N. Turner R. Paton A. (2021). The World Checklist of Vascular Plants, a continuously updated resource for exploring global plant diversity. Sci. Data 8, 215. 34389730 10.1038/s41597-021-00997-6 PMC8363670

[B25] Hagen O. Skeels A. Onstein R. E. Jetz W. Pellissier L. (2021). Earth history events shaped the evolution of uneven biodiversity across tropical moist forests. Proc. Natl. Acad. Sci. 118, e2026347118. doi: 10.1073/pnas.2026347118, PMID: 34599095 PMC8501849

[B26] Hayes P. E. Nge F. J. Cramer M. D. Finnegan P. M. Fu P. Hopper S. D. . (2021). Traits related to efficient acquisition and use of phosphorus promote diversification in Proteaceae in phosphorus-impoverished landscapes. Plant Soil 462, 67–88. doi: 10.1007/s11104-021-04886-0, PMID: 41810330

[B27] Heino J. Alahuhta J. Bini L. M. Cai Y. Heiskanen A. S. Hellsten S. . (2021). Lakes in the era of global change: moving beyond single-lake thinking in maintaining biodiversity and ecosystem services. Biol. Rev. 96, 89–106. doi: 10.1111/brv.12647, PMID: 32869448

[B28] Helmstetter A. J. Zenil-Ferguson R. Sauquet H. Otto S. P. Méndez M. Vallejo-Marin M. . (2023). Trait-dependent diversification in angiosperms: Patterns, models and data. Ecol. Lett. 26, 640–657. doi: 10.1111/ele.14170, PMID: 36829296

[B29] Hortal J. De Bello F. Diniz-Filho J. A. F. Lewinsohn T. M. Lobo J. M. Ladle R. J. (2015). Seven shortfalls that beset large-scale knowledge of biodiversity. Annu. Rev. ecology evolution systematics 46, 523–549. doi: 10.1146/annurev-ecolsys-112414-054400, PMID: 41139587

[B30] Huang E. Chen Y. Fang M. Zheng Y. Yu S. (2021). Environmental drivers of plant distributions at global and regional scales. Global Ecol. Biogeography 30, 697–709. doi: 10.1111/geb.13251, PMID: 41808597

[B31] Keil P. Chase J. M. (2019). Global patterns and drivers of tree diversity integrated across a continuum of spatial grains. Nat. Ecol. Evol. 3, 390–399. doi: 10.1038/s41559-019-0799-0, PMID: 30778185

[B32] Kier G. Kreft H. Lee T. M. Jetz W. Ibisch P. L. Nowicki C. . (2009). A global assessment of endemism and species richness across island and mainland regions. Proc. Natl. Acad. Sci. 106, 9322–9327. doi: 10.1073/pnas.0810306106, PMID: 19470638 PMC2685248

[B33] Kleiber C. Zeileis A. (2008). Applied econometrics with R ( Springer Science & Business Media).

[B34] Kreft H. Jetz W. (2007). Global patterns and determinants of vascular plant diversity. Proc. Natl. Acad. Sci. 104, 5925–5930. doi: 10.1073/pnas.0608361104, PMID: 17379667 PMC1851593

[B35] Lai J. Zou Y. Zhang S. Zhang X. Mao L. (2022). glmm. hp: an R package for computing individual effect of predictors in generalized linear mixed models. J. Plant Ecol. 15, 1302–1307. doi: 10.1093/jpe/rtac096

[B36] Lavin M. Herendeen P. S. Wojciechowski M. F. (2005). Evolutionary rates analysis of Leguminosae implicates a rapid diversification of lineages during the tertiary. Systematic Biol. 54, 575–594. doi: 10.1080/10635150590947131, PMID: 16085576

[B37] Lavin M. Schrire B. P. Lewis G. Pennington R. T. Delgado–Salinas A. Thulin M. . (2004). Metacommunity process rather than continental tectonic history better explains geographically structured phylogenies in legumes. Philos. Trans. R. Soc. London. Ser. B: Biol. Sci. 359, 1509–1522. doi: 10.1098/rstb.2004.1536, PMID: 15519969 PMC1693434

[B38] Le Provost G. Schenk N. V. Penone C. Thiele J. Westphal C. Allan E. . (2023). The supply of multiple ecosystem services requires biodiversity across spatial scales. Nat. Ecol. Evol. 7, 236–249. doi: 10.1038/s41559-022-01918-5, PMID: 36376602

[B40] Lewis G. Schrire B. MacKinder B. Lock M. (2005). Legumes of the World (Kew, UK: Royal Botanical Gardens).

[B41] Meyer C. Kreft H. Guralnick R. Jetz W. (2015). Global priorities for an effective information basis of biodiversity distributions. Nat. Commun. 6, 1–8. doi: 10.1038/ncomms9221, PMID: 26348291 PMC4569846

[B42] Meyer D. Zeileis A. Hornik K. Gerber F. Friendly M. Meyer M. D. (2020). Package ‘vcd’. R package version, Vol. 1. 1–4.

[B43] Nanglu K. de Carle D. Cullen T. M. Anderson E. B. Arif S. Castañeda R. A. . (2023). The nature of science: The fundamental role of natural history in ecology, evolution, conservation, and education. Ecol. Evol. 13, e10621. doi: 10.1002/ece3.v13.10, PMID: 37877102 PMC10591213

[B44] Owens H. L. Guralnick R. (2019). climateStability: An R package to estimate climate stability from time-slice climatologies. Biodiversity Inf. 14, 8–13. doi: 10.17161/bi.v14i0.9786

[B45] Oyebanji O. O. Onditi K. O. Azevedo J. A. Rahaingoson F. R. Nneji L. M. Adeleye M. A. . (2023). Biogeographic patterns and environmental drivers of species richness in the globally distributed Millettioid/Phaseoloid clade (Fabaceae, subfamily Papilionoideae). Front. Ecol. Evol. 11, 1231553. doi: 10.3389/fevo.2023.1231553, PMID: 41810299

[B46] Oyebanji O. O. Salako G. Nneji L. M. Oladipo S. O. Bolarinwa K. A. Chukwuma E. C. . (2021). Impact of climate change on the spatial distribution of endemic legume species of the Guineo-Congolian forest, Africa. Ecol. Indic. 122, 107282. doi: 10.1016/j.ecolind.2020.107282, PMID: 41810140

[B48] Peterson A. T. Soberón J. Sánchez-Cordero V. (1999). Conservatism of ecological niches in evolutionary time. Science 285, 1265–1267. doi: 10.1126/science.285.5431.1265, PMID: 10455053

[B2001] POWO . (2025). Available online at: https://powo.science.kew.org/ (Accessed February 15, 2025).

[B50] Qian H. Kessler M. Zhang J. Jin Y. Soltis D. E. Qian S. . (2023). Angiosperm phylogenetic diversity is lower in Africa than South America. Sci. Adv. 9, eadj1022. doi: 10.1126/sciadv.adj1022, PMID: 37967173 PMC10651126

[B49] Qian H. Qian S. (2023). Geographic patterns of taxonomic and phylogenetic β-diversity of angiosperm genera in regional floras across the world. Plant Diversity 45, 491–500. doi: 10.1016/j.pld.2023.07.008, PMID: 37936816 PMC10625901

[B51] Qian H. Qian S. Zhang J. Kessler M. (2024). Effects of climate and environmental heterogeneity on the phylogenetic structure of regional angiosperm floras worldwide. Nat. Commun. 15, 1079. doi: 10.1038/s41467-024-45155-9, PMID: 38316752 PMC10844608

[B52] Qiao X. Lamy T. Wang S. Hautier Y. Geng Y. White H. J. . (2023). Latitudinal patterns of forest ecosystem stability across spatial scales as affected by biodiversity and environmental heterogeneity. Global Change Biol. 29, 2242–2255. doi: 10.1111/gcb.16593, PMID: 36630490

[B54] Rahbek C. (2005). The role of spatial scale and the perception of large-scale species-richness patterns. Ecol. Lett. 8, 224–239. doi: 10.1111/j.1461-0248.2004.00701.x, PMID: 41808597

[B53] R Core Team (2022). R: A language and environment for statistical computing (Vienna Austria: R Foundation for Statistical Computing). Available online at: https://www.R-project.org/ (Accessed February 16, 2025).

[B55] Renner M. A. Foster C. S. Miller J. T. Murphy D. J. (2021). Phyllodes and bipinnate leaves of Acacia exhibit contemporary continental-scale environmental correlation and evolutionary transition-rate heterogeneity. Aust. Systematic Bot. 34, 595–608. doi: 10.1071/SB21009, PMID: 41161682

[B56] Roeble L. van Benthem K. J. Weigelt P. Kreft H. Knope M. L. Mandel J. R. . (2024). Island biogeography of the megadiverse plant family Asteraceae. Nat. Commun. 15, 7276. doi: 10.1038/s41467-024-51556-7, PMID: 39179568 PMC11343744

[B57] Sabatini F. M. Jiménez-Alfaro B. Jandt U. Chytrý M. Field R. Kessler M. . (2022). Global patterns of vascular plant alpha diversity. Nat. Commun. 13, 4683. doi: 10.1038/s41467-022-32063-z, PMID: 36050293 PMC9436951

[B58] Sandel B. Weigelt P. Kreft H. Keppel G. van der Sande M. T. Levin S. . (2020). Current climate, isolation and history drive global patterns of tree phylogenetic endemism. Global Ecol. Biogeography 29, 4–15. doi: 10.1111/geb.13001, PMID: 41808597

[B59] Siyum Z. G. (2020). Tropical dry forest dynamics in the context of climate change: syntheses of drivers, gaps, and management perspectives. Ecol. Processes 9, 1–16. doi: 10.1186/s13717-020-00229-6, PMID: 41810149

[B60] Smith S. A. Beaulieu J. M. Stamatakis A. Donoghue M. J. (2011). Understanding angiosperm diversification using small and large phylogenetic trees. Am. J. Bot. 98, 404–414. doi: 10.3732/ajb.1000481, PMID: 21613134

[B61] Song W. Li Y. Luo A. Su X. Liu Y. Luo Y. . (2024). The phylogenetic structure patterns of angiosperm species and their determinants in east Eurasia. Global Ecol. Biogeography 33, e13897. doi: 10.1111/geb.v33.10, PMID: 41808597

[B62] Stein A. Gerstner K. Kreft H. (2014). Environmental heterogeneity as a universal driver of species richness across taxa, biomes and spatial scales. Ecol. Lett. 17, 866–880. doi: 10.1111/ele.2014.17.issue-7, PMID: 24751205

[B63] Taylor A. Weigelt P. Denelle P. Cai L. Kreft H. (2023). The contribution of plant life and growth forms to global gradients of vascular plant diversity. New Phytol. 240, 1548–1560. doi: 10.1111/nph.v240.4, PMID: 37264995

[B64] Testolin R. Attorre F. Borchardt P. Brand R. F. Bruelheide H. Chytrý M. . (2021). Global patterns and drivers of alpine plant species richness. Global Ecol. Biogeography 30, 1218–1231. doi: 10.1111/geb.13297, PMID: 41808597

[B65] Tian Q. Stull G. W. Kellermann J. Medan D. Nge F. J. Liu S. Y. . (2024). Rapid in *situ* diversification rates in Rhamnaceae explain the parallel evolution of high diversity in temperate biomes from global to local scales. New Phytol. 241, 1851–1865. doi: 10.1111/nph.v241.4, PMID: 38229185

[B66] Tietje M. Antonelli A. Baker W. J. Govaerts R. Smith S. A. Eiserhardt W. L. (2022). Global variation in diversification rate and species richness are unlinked in plants. Proc. Natl. Acad. Sci. 119, e2120662119. doi: 10.1073/pnas.2120662119, PMID: 35767644 PMC9271200

[B67] Tietje M. Antonelli A. Forest F. Govaerts R. Smith S. A. Sun M. . (2023). Global hotspots of plant phylogenetic diversity. New Phytol. 240, 1636–1646. doi: 10.1111/nph.v240.4, PMID: 37496281

[B68] Tordoni E. Carmona C. P. Toussaint A. Tamme R. Pärtel M. (2024). Global patterns and determinants of multiple facets of plant diversity. Global Ecol. Biogeography 33, e13823. doi: 10.1111/geb.13823, PMID: 41808597

[B69] Venables W. N. Ripley B. D. (2002). Modern Applied Statistics with S. 4th ed. (New York: Springer).

[B70] Vitt P. Taylor A. Rakosy D. Kreft H. Meyer A. Weigelt P. . (2023). Global conservation prioritization for the Orchidaceae. Sci. Rep. 13, 6718. doi: 10.1038/s41598-023-30177-y, PMID: 37185616 PMC10130154

[B71] Wang Z. Wang T. Zhang X. Wang J. Yang Y. Sun Y. . (2024). Biodiversity conservation in the context of climate change: Facing challenges and management strategies. Sci. Total Environ., 173377. 38796025 10.1016/j.scitotenv.2024.173377

[B72] Weigelt P. Daniel Kissling W. Kisel Y. Fritz S. A. Karger D. N. Kessler M. . (2015). Global patterns and drivers of phylogenetic structure in island floras. Sci. Rep. 5, 12213. doi: 10.1038/srep12213, PMID: 26198002 PMC4510489

[B73] Weigelt P. König C. Kreft H. (2020). GIFT–A Global Inventory of Floras and Traits for macroecology and biogeography. J. Biogeography 47, 16–43. doi: 10.1111/jbi.13623, PMID: 41808597

[B74] Wiens J. J. Ackerly D. D. Allen A. P. Anacker B. L. Buckley L. B. Cornell H. V. . (2010). Niche conservatism as an emerging principle in ecology and conservation biology. Ecol. Lett. 13, 1310–1324. doi: 10.1111/j.1461-0248.2010.01515.x, PMID: 20649638

[B75] Xu W. B. Guo W. Y. Serra-Diaz J. M. Schrodt F. Eiserhardt W. L. Enquist B. J. . (2023). Global beta-diversity of angiosperm trees is shaped by Quaternary climate change. Sci. Adv. 9, eadd8553. doi: 10.1126/sciadv.add8553, PMID: 37018407 PMC10075971

[B76] Yang Y. Bian Z. Ren G. Liu J. Shrestha N. (2022). Niche conservatism limits the distribution of Medicago in the tropics. Ecography 2022, e06085.

[B77] Zhang R. Tian D. Wang J. Niu S. (2023). Critical role of multidimensional biodiversity in contributing to ecosystem sustainability under global change. Geogr. Sustainability 4, 232–243. doi: 10.1016/j.geosus.2023.05.002, PMID: 41810140

[B78] Zhao Y. Zhang R. Jiang K. W. Qi J. Hu Y. Guo J. . (2021). Nuclear phylotranscriptomics and phylogenomics support numerous polyploidization events and hypotheses for the evolution of rhizobial nitrogen-fixing symbiosis in Fabaceae. Mol. Plant 14, 748–773. doi: 10.1016/j.molp.2021.02.006, PMID: 33631421

