# Correction: Global patterns and drivers of species and genera richness of Fabaceae

**DOI:** 10.3389/fpls.2026.1821644

**Published:** 2026-03-12

**Authors:** Sazada Siddiqui

**Affiliations:** Department of Biology, College of Science, King Khalid University, Abha, Saudi Arabia

**Keywords:** Fabaceae, species richness, global patterns, drivers, biogeography

The affiliation “Department of Biology, College of Science, King Khalid University, Abha, Saudi Arabia” was erroneously given as “Department of Biology, College of Science, King Abdullah University, Abha, Saudi Arabia”.

The original version of this article has been updated.

