# Supplementary material for: Global patterns and drivers of species and genera richness of Fabaceae
Source: Front Plant Sci. 2025 Jul 21;16:1581814. doi: 10.3389/fpls.2025.1581814 (PMC12319032; doi:10.3389/fpls.2025.1581814)
Supplement: Supplementary file 1 [file DataSheet1.docx]

**Appendix:**

**Table S1.** List of explanatory variables and their abbreviated form

| **S. No.** | **Variable** | **Abbreviation** |
| --- | --- | --- |
| 1 | Annual Mean Temperature | Bio1 |
| 2 | Mean Diurnal Range (Mean of monthly (max temp - min temp)) | Bio2 |
| 3 | Isothermality (BIO2/BIO7) (×100) | Bio3 |
| 4 | Temperature Seasonality (standard deviation ×100) | Bio4 |
| 5 | Max Temperature of Warmest Month | Bio5 |
| 6 | Min Temperature of Coldest Month | Bio6 |
| 7 | Temperature Annual Range (BIO5-BIO6) | Bio7 |
| 8 | Mean Temperature of Wettest Quarter | Bio8 |
| 9 | Mean Temperature of Driest Quarter | Bio9 |
| 10 | Mean Temperature of Warmest Quarter | Bio10 |
| 11 | Mean Temperature of Coldest Quarter | Bio11 |
| 12 | Annual Precipitation | Bio12 |
| 13 | Precipitation of Wettest Month | Bio13 |
| 14 | Precipitation of Driest Month | Bio14 |
| 15 | Precipitation Seasonality (Coefficient of Variation) | Bio15 |
| 16 | Precipitation of Wettest Quarter | Bio16 |
| 17 | Precipitation of Driest Quarter | Bio17 |
| 18 | Precipitation of Warmest Quarter | Bio18 |
| 19 | Precipitation of Coldest Quarter | Bio19 |
| 20 | Geographical area | Area |
| 21 | Elevational range | ELR |
| 22 | Topographic position index | TPI |
| 23 | Terrain ruggedness index | TRI |
| 24 | Temperature stability | Tstab |
| 25 | Precipitation stability | Pstab |
| 26 | Temperature anomaly | Tanom |
| 27 | Precipitation anomaly | Panom |
| 28 | Velocity of temperature change | Tvel |


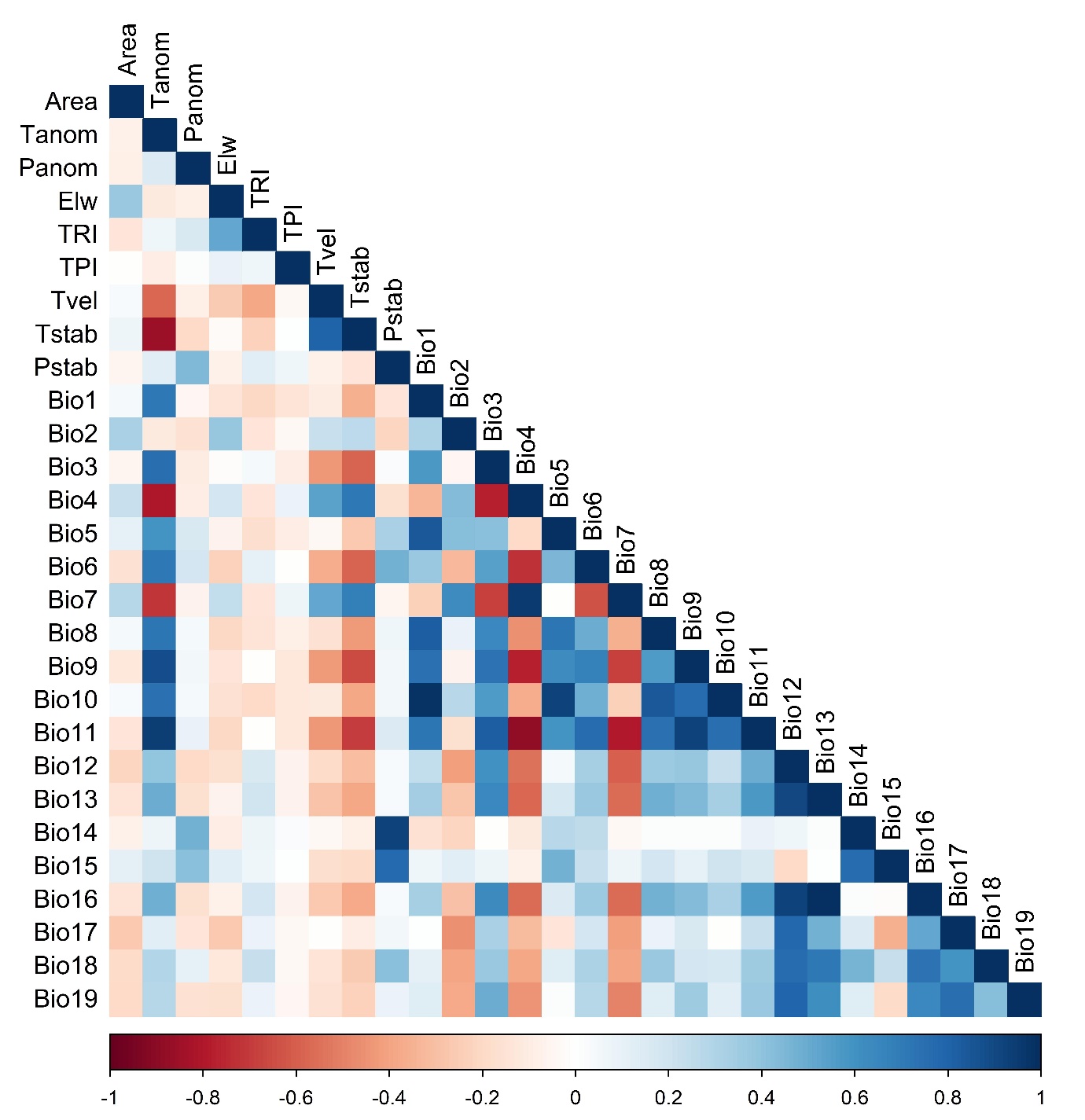
**Fig. S1** Correlation plot between the explanatory variables. See Table S1 for the full form of abbreviations used.
